# Prevalence and Associated Risk Factors of Acute Pancreatitis in Patients With Gallstones: A Cross-Sectional Study

**DOI:** 10.7759/cureus.84220

**Published:** 2025-05-16

**Authors:** Muhammad Saad Ul Hassan, Mariam Mobusher, Muhammad Hadi Mansoor, Chaudhary Adeel Ahmad, Iftikhar Ahsan, Muhammad Hamza, Muhammad Rasikh, Bilal Qammar

**Affiliations:** 1 General Surgery, DHQ Teaching Hospital, Gujranwala, Gujranwala, PAK; 2 General Surgery, Shalamar Hospital, Lahore, PAK; 3 General Surgery, Kamal Surgical Complex, Lodhran, PAK; 4 General Surgery, Rashid Latif Medical College, Lahore, PAK; 5 General Surgery, Lahore General Hospital, Lahore, PAK; 6 General Surgery, Muzaffarabad General Hospital, Muzaffarabad, PAK; 7 General Surgery, Jinnah Hospital, Lahore, PAK

**Keywords:** amylase, gallstones, lipase, pancreatic enzymes, pancreatitis

## Abstract

Introduction: Acute pancreatitis (AP) is a common and potentially life-threatening condition, with gallstones being a major etiology.

Objective: This study aims to determine how common AP is among patients with gallstones and to identify the associated risk factors, diagnostic laboratory markers, and potential complications.

Methodology: This cross-sectional study was conducted at Shalamar Hospital, Lahore, a tertiary care center, from June 2024 to January 2025. Data were collected from 165 patients presenting in the Department of Gastroenterology and General Surgery. Patients were included if they had gallstones confirmed by abdominal ultrasound and met the revised Atlanta criteria for AP, including clinical symptoms (e.g., epigastric pain, nausea) and laboratory findings (serum lipase/amylase ≥3× upper limit of normal).

Results: The prevalence of AP in patients with gallstones was 37.6% (62/165). Risk factors such as obesity (72%, 45/62), age (≥50 years, 72%, 45/62), and female gender (65%, 40/62) were significantly associated with the condition. Elevated serum amylase (225.4 ± 112.3 U/L) and lipase (317.8 ± 150.7 U/L), along with increased white blood cell (WBC) count (13.2 ± 4.6 x 10^9^/L), C-reactive protein (CRP) (45.7 ± 19.2 mg/L), and bilirubin levels (2.4 ± 1.6 mg/dL), were observed, particularly in severe cases of AP. Pancreatic necrosis occurred in 13% (8/62), organ failure in 8% (5/62), and systemic inflammatory response syndrome (SIRS) in 16% (10/62). Cholecystectomy was performed in 69% (43/62) of cases, and the mortality rate was 3.2% (2/62).

Conclusions: Gallstones are a major cause of AP, with obesity, age, and female gender being key risk factors. Elevated laboratory markers correlate with the severity of the condition and can aid in disease assessment.

## Introduction

Acute pancreatitis (AP) is a sudden inflammatory condition of the pancreas associated with significant morbidity and mortality. Gallstones are the most frequent cause of AP, with demographic changes such as increasing obesity rates, an aging population, and evolving dietary habits contributing to their rising prevalence [1]. Between one-third and 40% of all AP cases are attributed to gallstone disease [2,3]. Identifying the prevalence and associated risk factors in patients with gallstones enables better recognition of high-risk individuals and informs the development of preventive strategies [2].

The mechanism of gallstone-induced AP involves obstruction of the common bile duct or pancreatic duct, leading to increased ductal pressure and subsequent activation of pancreatic enzymes, particularly trypsin [4,5]. This premature enzyme activation results in autodigestion of pancreatic tissue and triggers local inflammation. In some cases, gallstones can also facilitate the reflux of bile into the pancreatic duct, further activating pancreatic enzymes and amplifying inflammation [5]. Notably, the development of AP in patients with gallstones remains relatively uncommon, suggesting that other contributory risk factors must be present [6].

Several clinical and population-specific risk factors have been identified in gallstone-related AP. Obesity is particularly significant, as it not only promotes the formation of gallstones but also exacerbates the severity of pancreatitis symptoms [7]. High-fat diets, sedentary behavior, and metabolic syndrome are likewise associated with a greater incidence of both gallstones and AP [7]. Alcohol abuse increases the likelihood of both conditions by promoting pancreatic inflammation and structural damage [8]. Age also plays a role; individuals aged 40 years and older show increased susceptibility to gallstones and, consequently, a higher risk of developing AP [8]. Additionally, women are more prone to gallstones due to hormonal influences, especially during pregnancy, postpartum periods, and with the use of oral contraceptives [9].

Genetic predispositions also influence susceptibility to both gallstones and AP. Mutations in the cystic fibrosis transmembrane conductance regulator (CFTR) gene and genes affecting bile acid metabolism are linked to a higher risk of both conditions [10]. Hereditary pancreatitis, often caused by mutations such as PRSS1, represents a distinct genetic condition that can lead to recurrent or chronic pancreatitis independent of gallstone presence [11]. The interplay between genetic factors and environmental exposures appears to significantly influence individual vulnerability to disease onset and progression [4].

This study aims to determine how common AP is among patients with gallstones and to identify the associated risk factors, diagnostic laboratory markers, and potential complications.

## Materials and methods

Methodology

This cross-sectional study was conducted at Shalamar Hospital, Lahore, a tertiary care center, from June 2024 to January 2025. Data were collected from 165 patients presenting in the Department of Gastroenterology and General Surgery. Patients were included if they had gallstones confirmed by abdominal ultrasound and met the revised Atlanta criteria for AP, including clinical symptoms (e.g., epigastric pain, nausea) and laboratory findings (serum lipase/amylase ≥3× upper limit of normal).

Sample size

A consecutive sampling approach was used, enrolling all eligible patients sequentially during the study period to minimize selection bias. A sample size of 165 patients was calculated using G*Power 3.1 (The G*Power Team, Germany), assuming a 30% prevalence of AP in gallstone patients [2], 80% power, and 5% margin of error.

Inclusion criteria

The study enrolled patients aged over 18 years with a confirmed diagnosis of gallstones confirmed by abdominal ultrasound and who met the revised Atlanta criteria for AP, including clinical symptoms (e.g., epigastric pain, nausea) and laboratory findings (serum lipase/amylase ≥3× upper limit of normal). Participants were required to exhibit an acute onset of pancreatitis, defined by elevated serum amylase and lipase levels exceeding the upper limit of normal, accompanied by compatible clinical signs and symptoms, such as epigastric pain, nausea, vomiting, or radiographic evidence of pancreatic inflammation. Additionally, all included individuals provided written informed consent to participate in the research, ensuring their willingness to adhere to the study protocol and follow-up requirements.

Exclusion criteria

Individuals were excluded if they had a prior history of chronic pancreatitis or if their acute pancreatitis was attributed to alternative etiologies, such as excessive alcohol consumption, hypertriglyceridemia (serum triglycerides >500 mg/dL), or medication-induced pancreatitis. Patients with incomplete clinical records, including missing laboratory or imaging data essential for diagnosis or analysis, were also excluded. Furthermore, those with severe comorbidities such as advanced cardiac, renal, or respiratory diseases that could independently influence clinical outcomes or complicate the interpretation of results were omitted from the study cohort to minimize confounding factors.

Data collection

Data were collected from the hospital's patient records, and detailed medical history was obtained from each participant through a structured questionnaire. Data were double-checked for consistency and completeness by two independent reviewers. Questionnaires were pilot-tested on 10 patients for clarity and reliability before implementation. Data included demographic information, age, gender, clinical characteristics, BMI, presence of diabetes, hypertension, hyperlipidemia, and specific details about gallstones (size, location, and number of stones) and pancreatitis (severity, presence of complications such as pancreatic necrosis or organ failure). Lifestyle factors such as alcohol consumption, dietary habits, and physical activity were also noted. All participants were also evaluated for additional risk factors that might contribute to acute pancreatitis, including a family history of gallstones or pancreatitis. Risk factors associated with acute pancreatitis in gallstone patients were identified through a detailed questionnaire administered to the participants.

Data analysis

Data were analyzed using Statistical Product and Service Solutions (SPSS, version 26; IBM SPSS Statistics for Windows, Armonk, NY). Descriptive statistics, including frequency distributions, percentages, and means with standard deviations (SD), were used to determine the prevalence of acute pancreatitis among patients with gallstones. To assess the relationship between various risk factors and the development of acute pancreatitis, chi-square tests were employed for categorical variables, and t-tests were used for continuous variables. Effect size was calculated using Cramer’s V. P-values < 0.05 were considered statistically significant.

## Results

A total of 165 patients were added to the study, with an average age of 55.4 ± 12.7 years. Among the patients, 105 (63.6%) were female, and 60 (36.4%) were male. Obesity was present in 74 (45%) of patients, while comorbidities such as diabetes, hypertension, and hyperlipidemia were observed in 63 (38%), 83 (50%), and 69 (42%) of the cohort, respectively. Gallstones were found to be multiple in 91 (55%) of patients, with 66 (40%) having stones larger than 2 cm, and the average size of the stones was 1.5 cm ± 0.5 cm (Table 1).

**Table 1:** Demographic and Clinical Characteristics of the Study Population SD: Standard Deviation, BMI: Body Mass Index

Characteristic	Value
Total Patients	165
Age (Mean ± SD)	55.4 ± 12.7
Gender (Female)	105 (63.6%)
Gender (Male)	60 (36.4%)
Obesity (BMI > 30)	74 (45%)
Diabetes Mellitus	63 (38%)
Hypertension	83 (50%)
Hyperlipidemia	69 (42%)
Number of Gallstones (Multiple)	91 (55%)
Gallstone Size (>2 cm)	66 (40%)
Gallstone Size (Mean ± SD)	1.5 cm ± 0.5 cm

Serum amylase and lipase were elevated in 61 (98%) and 59 (95%) patients, with mean values of 225.4 ± 112.3 U/L and 317.8 ± 150.7 U/L, respectively, indicating the acute nature of pancreatitis. The white blood cell count (WBC) was elevated in 52 (84%) patients, with a mean value of 13.2 ± 4.6 x10^9^/L, reflecting the inflammatory response. C-reactive protein (CRP) was elevated in 55 (89%) of patients, with a mean of 45.7 ± 19.2 mg/L, suggesting systemic inflammation, often preceding clinical deterioration. Liver function tests showed elevated alanine aminotransferase (ALT) and aspartate aminotransferase (AST) in 36 (58%) patients, with mean values of 120.5 ± 58.3 U/L and 100.3 ± 50.1 U/L, respectively (Table 2).

**Table 2:** Laboratory Findings in Patients With Acute Pancreatitis SD: Standard Deviation, ALT: Alanine Aminotransferase, AST: Aspartate Aminotransferase

Laboratory Test	Normal Range	Patients with Acute Pancreatitis (%)	Mean Value ± SD
Serum Amylase (U/L)	30-110	98% (61/62)	225.4 ± 112.3
Serum Lipase (U/L)	23-300	95% (59/62)	317.8 ± 150.7
White Blood Cell Count (WBC) (x10^9^/L)	4.0-10.5	84% (52/62)	13.2 ± 4.6
C-Reactive Protein (CRP) (mg/L)	< 10	89% (55/62)	45.7 ± 19.2
Liver Function Tests (ALT, AST) (U/L)	10-40	58% (36/62)	ALT: 120.5 ± 58.3; AST: 100.3 ± 50.1
Bilirubin (Total) (mg/dL)	0.1-1.2	49% (30/62)	2.4 ± 1.6

The prevalence of AP in patients with gallstones was found to be 62 (37.6%). Among those with AP, 69 (42%) experienced mild forms, 65 (39%) had moderate cases, and 31 (19%) developed severe AP (Table 3).

**Table 3:** Prevalence of Acute Pancreatitis in Patients With Gallstones

Characteristic	Value
Prevalence of Acute Pancreatitis	62 (37.6%)
Acute Pancreatitis (Mild)	69 (42%)
Acute Pancreatitis (Moderate)	65 (39%)
Acute Pancreatitis (Severe)	31 (19%)

**Figure 1:**
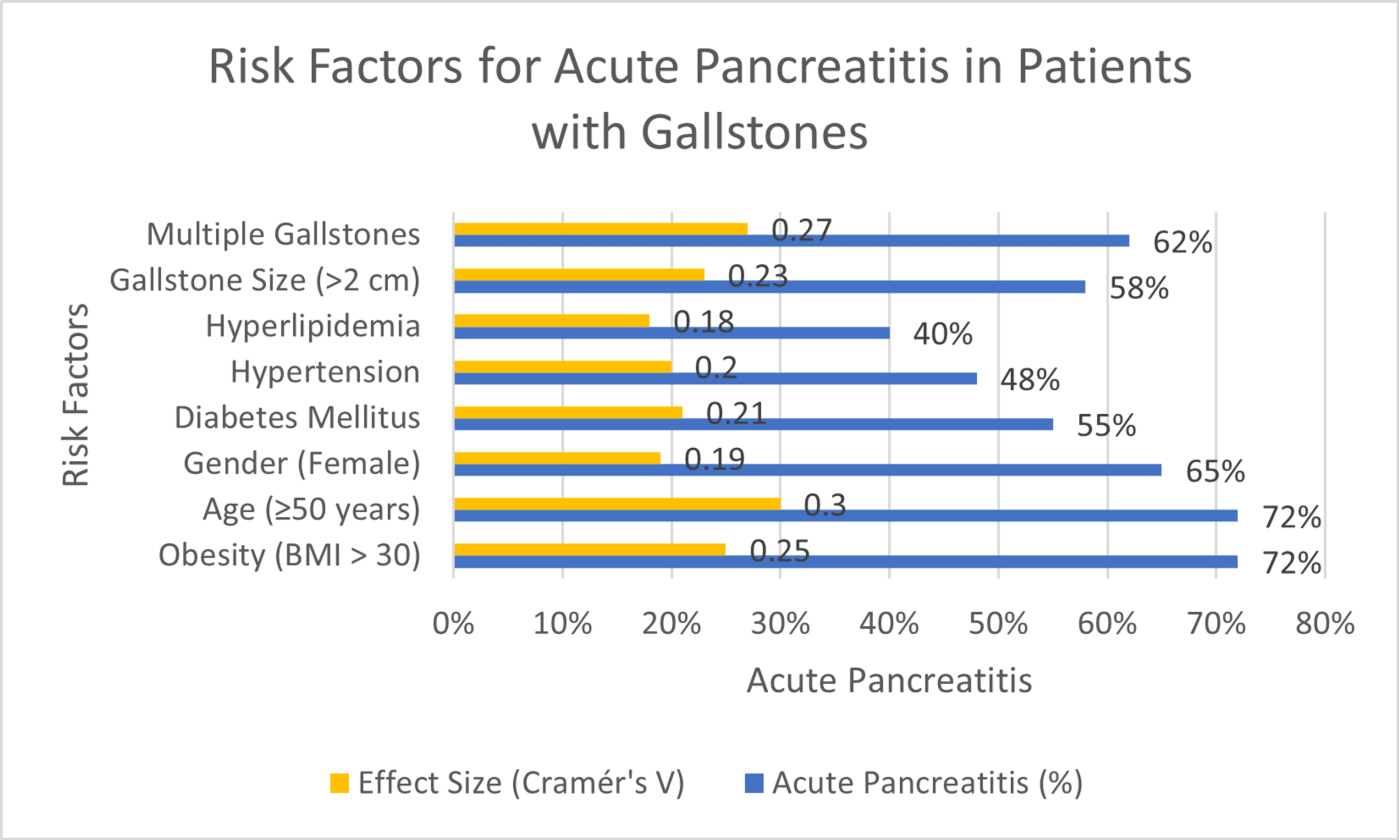
Risk Factors for Acute Pancreatitis in Patients With Gallstones

Obesity (BMI >30) and age (≥50 years) were strongly linked to a higher incidence of AP, with 45 (72%) cases occurring in these groups. Chi-square values for both risk factors were 10.44 and 12.34, respectively, with p-values of less than 0.05 and effect sizes (Cramér's V) of 0.25 and 0.30, indicating medium to large associations. Gender (female) was also a significant risk factor, with 40 (65%) women developing AP (chi-square = 5.75, p < 0.05, effect size = 0.19). Other risk factors, such as diabetes (34, 55%), hypertension (30, 48%), hyperlipidemia (25, 40%), and larger gallstones (>2 cm; 36, 58%), also showed significant associations with AP, with chi-square values ranging from 6.99 to 9.02 and p-values less than 0.05 (Table 4, Figure 1).

**Table 4:** Risk Factors for Acute Pancreatitis in Patients With Gallstones P-values < 0.05 were considered statistically significant.

Risk Factor	Acute Pancreatitis (%)	Chi-Square Value	p-value	Effect Size (Cramér's V)
Obesity (BMI > 30)	45 (72%)	10.44	< 0.05	0.25
Age (≥50 years)	45 (72%)	12.34	< 0.01	0.30
Gender (Female)	40 (65%)	5.75	< 0.05	0.19
Diabetes Mellitus	34 (55%)	8.64	< 0.05	0.21
Hypertension	30 (48%)	7.82	< 0.05	0.20
Hyperlipidemia	25 (40%)	6.99	< 0.05	0.18
Gallstone Size (>2 cm)	36 (58%)	9.02	< 0.01	0.23
Multiple Gallstones	39 (62%)	11.67	< 0.01	0.27

The complications and outcomes observed in patients with AP in this study were as follows: pancreatic necrosis occurred in 8/62 (13%) of patients, with a chi-square value of 3.55, p < 0.05, and an effect size of 0.17, indicating a small association. Organ failure was observed in 5/62 (8%) of patients, with a chi-square value of 4.22, p < 0.05, and an effect size of 0.16, showing a small effect. Systemic inflammatory response syndrome (SIRS) was seen in 10/62 (16%) of patients, with a chi-square value of 6.30, p < 0.05, and an effect size of 0.19, indicating a moderate association. The high frequency of cholecystectomy (gallbladder removal), 43/62 (69%), highlights proactive management strategies to prevent recurrence, aligning with recent surgical guidelines, with a chi-square value of 7.24, p < 0.05, and an effect size of 0.21, suggesting a medium-strength association (Table 5).

**Table 5:** Complications and Management Outcomes in Patients with Acute Pancreatitis P-values < 0.05 were considered statistically significant.

Complication/Outcome	Number of Patients *n/N *(%)	Chi-Square Value	p-value	Effect Size (Cramér's V)
Pancreatic Necrosis	8/62 (13%)	3.55	< 0.05	0.17
Organ Failure	5/62 (8%)	4.22	< 0.05	0.16
Systemic Inflammatory Response Syndrome (SIRS)	10/62 (16%)	6.30	< 0.05	0.19
Cholecystectomy (Gallbladder Removal)	43/62 (69%)	7.24	< 0.05	0.21
Mortality	2/62 (3.2%)	-	-	-

Serum amylase and lipase levels were higher in severe cases, suggesting a correlation with enzymatic injury and tissue necrosis, with chi-square values of 9.42 (p < 0.05) and 7.91 (p < 0.05), respectively, indicating moderate associations. White blood cell count, CRP, liver function tests, and bilirubin levels also increased with severity, with smaller effect sizes ranging from 0.19 to 0.28 (Table 6).

**Table 6:** Association of Laboratory Findings With the Severity of Acute Pancreatitis ALT: Alanine Aminotransferase, AST: Aspartate Aminotransferase P-values < 0.05 were considered statistically significant.

Laboratory Test	Mild Acute Pancreatitis (%)	Moderate Acute Pancreatitis (%)	Severe Acute Pancreatitis (%)	Chi-Square Value	p-value	Effect Size (Cramér's V)
Serum Amylase (U/L)	240.5 ± 98.4	235.2 ± 112.0	350.2 ± 145.1	9.42	< 0.05	0.28
Serum Lipase (U/L)	310.5 ± 145.3	298.4 ± 143.2	410.1 ± 160.5	7.91	< 0.05	0.25
White Blood Cell Count (WBC) (x10^9^/L)	12.1 ± 4.2	13.6 ± 5.1	16.2 ± 5.8	6.67	< 0.05	0.22
C-Reactive Protein (CRP) (mg/L)	40.3 ± 18.5	48.7 ± 21.3	55.3 ± 22.7	5.83	< 0.05	0.21
Liver Function Tests (ALT, AST) (U/L)	110.2 ± 50.1	115.6 ± 58.4	130.5 ± 60.2	4.53	< 0.05	0.19
Bilirubin (Total) (mg/dL)	1.8 ± 0.9	2.2 ± 1.1	3.1 ± 1.4	5.02	< 0.05	0.20

## Discussion

This study aimed to explore the prevalence and associated risk factors of AP in patients with gallstones, emphasizing the relationship between demographic, clinical, and laboratory findings with the development and severity of the condition. These research findings demonstrate that AP develops through multiple factors when gallstones are present, while identifying essential risk factors that help guide medical decisions. This study demonstrated AP occurrence in 62 (37.6%) patients with gallstones, matching previous research about gallstones as one of the main causes of AP in 30-40% of cases [2,3,6].

A significant observation in this study was the association between obesity (BMI >30) and increased AP risk, where 72% of patients with AP were obese. These results support the findings by Weiss et al., who emphasize the role of adipose tissue-driven inflammation and metabolic dysfunction in exacerbating pancreatic injury [10]. Obesity contributes to gallstone formation by increasing biliary cholesterol saturation and gallbladder hypomotility, both of which are well-documented in the pathogenesis of gallstone-induced AP [9]. Additionally, advanced age (≥50 years) and female gender emerged as prominent risk factors. This is consistent with epidemiological data that highlight age-related changes in biliary composition and motility, as well as hormonal influences in women, particularly during pregnancy and with estrogen exposure, which elevate cholesterol secretion into bile [9]. The predominance of women among AP patients (65%) in our study is similar to findings by Tess et al. and the American Gastroenterological Association [6,9]. The role of comorbidities was also significant. Diabetes mellitus (55%), hypertension (48%), and hyperlipidemia (40%) were more prevalent in patients with AP. These findings reinforce the view that AP is part of a broader metabolic dysfunction landscape, where insulin resistance and hypertriglyceridemia contribute to both gallstone formation and pancreatic injury [10].

Efforts made in the laboratory yielded essential data to determine the clinical seriousness of AP [12]. The diagnosis of AP matched with elevated serum amylase and lipase results, yet this severity index increased with serious pancreatitis manifestations. Laboratory findings in our study, particularly elevated serum amylase, lipase, CRP, and bilirubin levels, correlated well with the severity of AP. These biomarkers are widely used to assess inflammation, enzymatic activity, and hepatobiliary involvement [1,13]. Notably, higher enzyme levels were strongly associated with severe AP cases, echoing the observations of Peery et al., who reported that enzyme trends can help predict pancreatic necrosis and hospital course [13]. One critical outcome of this study was the complication rate among AP patients, which included pancreatic necrosis (13%), organ failure (8%), and SIRS (16%). These complications were more likely in patients with significantly elevated inflammatory markers, supporting the current understanding that systemic inflammation and multiorgan dysfunction are driven by both local pancreatic injury and a proinflammatory milieu [11,14]. Patients experiencing these complications had higher chances of death during their hospital stay, with prolonged hospitalization time [14].

Our study also reported a cholecystectomy rate of 69% among AP patients. Early cholecystectomy is recommended to prevent recurrent attacks of gallstone-related AP, especially in mild-to-moderate cases [7]. The remaining 31% who did not undergo surgery could represent cases of delayed intervention, contraindications, or patient refusal, warranting further investigation. In a meta-analysis by Moody et al., early cholecystectomy was shown to significantly reduce recurrence without increasing surgical risks [7]. The research findings emphasize that effective management must incorporate active treatment of diabetes along with hypertension and hyperlipidemia in addition to standard pancreatitis procedures [15,16].

This study’s findings hold important clinical implications. Identifying high-risk profiles, especially obese, older female patients with metabolic comorbidities, allows for targeted screening and early intervention, such as dietary modification, lifestyle changes, or elective cholecystectomy before complications arise. Furthermore, monitoring laboratory markers, such as CRP and bilirubin, in these high-risk groups could serve as early indicators of disease escalation. Despite these strengths, this study has limitations that must be acknowledged. Because the study uses a cross-sectional design, it hinders the capability to prove cause-and-effect relationships between risk elements and pancreatitis development. The conducting of research in a single institution reduces its ability to provide results applicable to diverse populations. The evaluation of gallstone-related AP requires additional research using larger collection sites combined with extended patient follow-up to validate results and measure extended patient responses.

## Conclusions

It is concluded that acute pancreatitis is a significant complication among patients with gallstones, with a prevalence of 37.6% in this study. Obesity, older age, and female gender were identified as key risk factors for the development of acute pancreatitis in these patients. Laboratory findings, particularly elevated serum amylase, lipase, CRP, and bilirubin levels, were found to correlate with the severity of the disease. The presence of comorbid conditions such as diabetes, hypertension, and hyperlipidemia further exacerbates the risk and severity of pancreatitis.

## References

[1] James TW, Crockett SD (2018). Management of acute pancreatitis in the first 72 hours. Curr Opin Gastroenterol.

[2] Kundumadam S, Fogel EL, Gromski MA (2021). Gallstone pancreatitis: general clinical approach and the role of endoscopic retrograde cholangiopancreatography. Korean J Intern Med.

[3] Kim J (2017). Training in endoscopy: endoscopic retrograde cholangiopancreatography. Clin Endosc.

[4] Buxbaum JL, Abbas Fehmi SM, Sultan S (2019). ASGE guideline on the role of endoscopy in the evaluation and management of choledocholithiasis. Gastrointest Endosc.

[5] Muangkaew P, Kamalaporn P, Mingphruedhi S, Rungsakulkij N, Suragul W, Vassanasiri W, Tangtawee P (2020). Outcomes of delayed endoscopic retrograde cholangiopancreatography in patients with acute biliary pancreatitis with cholangitis. Asian J Surg.

[6] Vege SS, DiMagno MJ, Forsmark CE, Martel M, Barkun AN (2018). Initial medical treatment of acute pancreatitis: American Gastroenterological Association institute technical review. Gastroenterology.

[7] Moody N, Adiamah A, Yanni F, Gomez D (2019). Meta-analysis of randomized clinical trials of early versus delayed cholecystectomy for mild gallstone pancreatitis. Br J Surg.

[8] García de la Filia Molina I, García García de Paredes A, Martínez Ortega A (2019). Biliary sphincterotomy reduces the risk of acute gallstone pancreatitis recurrence in non-candidates for cholecystectomy. Dig Liver Dis.

[9] Tess A, Freedman SD, Kent T, Libman H (2019). How would you treat this patient with gallstone pancreatitis?: grand rounds discussion from Beth Israel Deaconess Medical Center. Ann Intern Med.

[10] Weiss FU, Laemmerhirt F, Lerch MM (2019). Etiology and risk factors of acute and chronic pancreatitis. Visc Med.

[11] Frost F, Schlesinger L, Wiese ML (2022). Infection of (peri-)pancreatic necrosis is associated with increased rates of adverse events during endoscopic drainage: a retrospective study. J Clin Med.

[12] Frost F, Khaimov V, Senz V (2024). The composition of the stent microbiome is associated with morbidity and adverse events during endoscopic drainage therapy of pancreatic necroses and pseudocysts. Front Med (Lausanne).

[13] Peery AF, Crockett SD, Murphy CC (2022). Burden and cost of gastrointestinal, liver, and pancreatic diseases in the United States: update 2021. Gastroenterology.

[14] van Brunschot S, van Grinsven J, van Santvoort HC (2018). Endoscopic or surgical step-up approach for infected necrotising pancreatitis: a multicentre randomised trial. Lancet.

[15] Khaimov V, Frost F, Lerch MM, Senz V, Grabow N, Schmitz KP (2020). Systematic microscopic analysis of retrieved stents from a patient with pancreatic necrosis. Curr Direct Biomed Eng.

[16] Zhu Y, He C, Li X (2019). Gut microbiota dysbiosis worsens the severity of acute pancreatitis in patients and mice. J Gastroenterol.

